# Inferences about spatiotemporal variation in dengue virus transmission are sensitive to assumptions about human mobility: a case study using geolocated tweets from Lahore, Pakistan

**DOI:** 10.1140/epjds/s13688-018-0144-x

**Published:** 2018-06-11

**Authors:** Moritz U. G. Kraemer, D. Bisanzio, R. C. Reiner, R. Zakar, J. B. Hawkins, C. C. Freifeld, D. L. Smith, S. I. Hay, J. S. Brownstein, T. Alex Perkins

**Affiliations:** 1 Department of Pediatrics, Harvard Medical School, Boston, USA; 2 Computational Epidemiology Lab, Boston Children’s Hospital, Boston, USA; 3 Department of Zoology, University of Oxford, Oxford, UK; 4 RTI International, Washington, USA; 5 Center for Tropical Diseases, Sacro Cuore-Don Calabria Hospital, Negrar, Italy; 6 Institute for Health Metrics and Evaluation, University of Washington, Seattle, USA; 7 Department of Public Health, University of Punjab, Lahore, Pakistan; 8 College of Computer and Information Science, Northeastern University, Boston, USA; 9 Sanaria Institute for Global Health and Tropical Medicine, Rockville, USA; 10 Department of Biological Sciences and Eck Institute for Global Health, University of Notre Dame, Notre Dame, USA

**Keywords:** Big data, Disease dynamics, Geo-located tweets, Gravity model, Human mobility, Radiation model, Spatiotemporal analysis, Twitter data

## Abstract

Billions of users of mobile phones, social media platforms, and other technologies generate an increasingly large volume of data that has the potential to be leveraged towards solving public health challenges. These and other big data resources tend to be most successful in epidemiological applications when utilized within an appropriate conceptual framework. Here, we demonstrate the importance of assumptions about host mobility in a framework for dynamic modeling of infectious disease spread among districts within a large urban area. Our analysis focused on spatial and temporal variation in the transmission of dengue virus (DENV) during a series of large seasonal epidemics in Lahore, Pakistan during 2011–2014. Similar to many directly transmitted diseases, DENV transmission occurs primarily where people spend time during daytime hours, given that DENV is transmitted by a day-biting mosquito. We inferred spatiotemporal variation in DENV transmission under five different assumptions about mobility patterns among ten districts of Lahore: no movement among districts, movement following patterns of geo-located tweets, movement proportional to district population size, and movement following the commonly used gravity and radiation models. Overall, we found that inferences about spatiotemporal variation in DENV transmission were highly sensitive to this range of assumptions about intra-urban human mobility patterns, although the three assumptions that allowed for a modest degree of intra-urban mobility all performed similarly in key respects. Differing inferences about transmission patterns based on our analysis are significant from an epidemiological perspective, as they have different implications for where control efforts should be targeted and whether conditions for transmission became more or less favorable over time.

**Electronic Supplementary Material:**

The online version of this article (10.1140/epjds/s13688-018-0144-x) contains supplementary material.

## Introduction

The spread and transmission dynamics of human infectious diseases are shaped extensively by human behavior [18]. Pathogen transmission depends on human contact patterns and tends to accelerate in highly connected areas with high population size and frequent travel [23]. Relevant population interactions between areas can be the result of daily commuting to the commercial center of a city and back [32, 50], visiting relatives or friends [27], religious or cultural activities [15], or many other reasons. Generally, urban travel is characterized by extensive daily activity, as work activities do not typically take place at the same places where people live [19]. Dynamic human movement patterns in cities can be inferred using a variety of data sources such as census data, mobile phone data, or social media data [28]. Passive data collection from social media platforms now offer timely, high resolution estimates of spatiotemporal patterns of human mobility [4, 5, 28, 32]. All of these movement types have the potential to shape infectious disease transmission dynamics, potentially in different ways depending on the mode of transmission (e.g., by direct contact or through a mosquito vector).

Specifically in the context of urban transmission, the importance of spatial heterogeneity in drivers of transmission is well-documented [40, 47, 54]. Some districts of a city may have considerably higher likelihood of infection as a result of, for example, higher mosquito densities (e.g., malaria [53], dengue [56]), yet each district contributes to transmission in any other district, not just within its own boundaries, due largely to human travel [11, 31, 34]. Such considerations can become highly relevant when human mobility is high, as observed in large urban areas [12] and can critically inform how resources to control and eliminate disease should be allocated [9, 11]. Understanding this interaction between human mobility, spatial variation in drivers of transmission, and control measures is important to know where control measures will be most impactful, as dengue [48], chikungunya [14, 49], yellow fever [21, 22], and Zika [30] continue to cause large urban outbreaks to control their spread and limit the burden caused by these viruses. An important feature that they have in common is that they are all transmitted by the *Aedes* mosquitoes, which are active during daytime hours when human mobility is high [54].

In this study, we examined a series of seasonal dengue epidemics in an urban setting that occurred between 2011 and 2014 in Lahore, Pakistan; no major epidemic had been recorded before that date [24]. Dengue virus (DENV) is a flavivirus transmitted between humans primarily by the *Aedes aegypti* mosquito [51]. Dengue burden is enormous and it has increased substantially in recent decades [6]. The distribution of *Ae. aegypti* is now larger than it has ever known to be [25], and the viruses it transmits have been expanding too as a result [29, 33], leading to expanding ranges or changes in the epidemiology of Zika, chikungunya, and yellow fever.

To enhance our understanding of urban transmission dynamics of infectious diseases and to evaluate the importance of assumptions of the spatial configuration of cities, we here use human mobility models and estimates derived from the social network platform Twitter to compare inferences about spatiotemporal variation in transmission patterns and determine how sensitive these inferences are to different assumptions about patterns of intra-urban human mobility. Little sensitivity of these inferences would suggest that analyses could proceed with business as usual assumptions, whereas strong sensitivity would point to a need for more careful consideration of human mobility data within analyses of infectious disease dynamics, even at the granularity of intra-urban scales.

## Material & methods

*Epidemiological data*: We obtained individual dengue case data from Lahore, Pakistan, and aggregated to the town level (an administrative subdivision of the city, $n = 10$) on a weekly basis from January 1, 2011 to December 31, 2014. We refer to the number of dengue cases reported in town *i* in week *t* as $I _{i,t}$. Data were provided by the Health Department, Pakistan, and were processed from their original line list form. In total, 35,348 confirmed and suspected cases were recorded in Lahore. Roughly 18,020 of those occurred during the 2011 epidemic alone. Details of the geo-positioning procedure are described in detail in Kraemer et al. [26].

*Human mobility data and models*: To quantify human mobility patterns, we used openly available data from Twitter through its API. Our database consists of tweets made in Lahore from January 1, 2011 through June 30, 2015. Specifically, the tweets were gathered by querying the free streaming API for a bounding box of $[-180, 180]$ longitude and $[-90, 90]$ latitude, so all tweets with geographic coordinates match. The results are limited by Twitter to 1% of total tweet volume. We then filtered the database to only include tweets sent within the city of Lahore, Pakistan. The penetration of Twitter users with geo-located information amounts to about 1% of the total population in the study period, similar to previous estimates [28]. Other information included the user’s unique ID. We associated each user with a town of residence according to which town they sent most tweets from during night hours defined as 9pm–7am.

To use the tweets to summarize mobility patterns of residents of the 10 different towns, we computed a single matrix *H* that contained the proportion of tweets made in town *j* by residents of town *i*, where *i* and *j* refer to the row and column of *H*, respectively. Thus, the rows of *H* sum to 1, and the columns of *H* sum to values somewhat less than or greater than 1. Due to the somewhat limited number of tweets available from users in a given town during a given time period and because there was no obvious seasonality in the data, we did not make use of temporally disaggregated Twitter data in our transmission model.

In addition to the *H* matrix based on tweets, we constructed four alternative *H* matrices that span a wide range of assumptions about human mobility commonly used in infectious disease modeling. At one extreme, we constructed an *H* matrix following the ideal free assumption that movements between all locations occur proportional to population size. At the opposite extreme, we constructed an *H* matrix consistent with an assumption of no movement between towns. Just as the *H* matrix based on tweets represents an intermediate assumption between these two extremes, we formulated two additional *H* matrices based on commonly used models of human mobility; the gravity model [60] and the radiation model [50]. We applied these models to data about the distance between town centroids and town population sizes. This produced values of fluxes between *i* and *j* but did not produce an estimate of the magnitude of time spent in *i* by residents of *i*. To work around this gap in the predictions of these models, we used the diagonal of the tweet-based *H* matrix as the diagonal for these two *H* matrices. For the off-diagonal elements, we normalized the fluxes out of *i* predicted by the gravity and radiation models and multiplied those terms by $1-H _{i,i}$. Numerical values of all five *H* matrices are provided in Tables S1–S5 (Additional file 1).

*Mobility-based transformation of incidence data*: Our analysis is premised on the distinction between the location where an individual resides and the locations where she or he spends time. DENV is transmitted by the urban-adapted mosquito *Ae. aegypti*, which engages in the majority of its blood feeding activity during daytime hours [1]. Because this means that transmission is expected to occur mainly where people spend time during the day rather than where they reside [55], we transformed the ten residence-based incidence time series $I _{i,t}$ to ten mobility-based incidence time series $\tilde{I}_{i,t}$. The latter contains the incidence of cases acquired in town *i* in week *t* under a given assumption about mobility patterns defined by *H* and is calculated according to $\tilde{I}_{i,t} = \sum_{j} H_{j,i} I_{j,t}$. We examined a total of five different interpretations of $\tilde{I}_{i,t}$ corresponding to the five different assumptions about human mobility patterns quantified by five different *H* matrices, as described in the previous section.

*Transmission model*: We used a spatial TSIR framework to model the dynamics of $\tilde{I}_{i,t}$ in the ten towns of Lahore during 2011–2014. Consistent with the assumptions about mobility used to define $\tilde{I}_{i,t}$, we defined the effective population size of town *i* during daytime hours as $\tilde{N}_{i} = \sum_{j} H_{j,i} N_{j}$. We are not aware of any significant DENV transmission activity in Lahore prior to 2011, so we assumed that the effective number of susceptible individuals in town *i* during daytime hours was $\tilde{S}_{i,1} = \tilde{N}_{i}$ during the first week of January, 2011. Thenceforth, the susceptible population was depleted as new cases arose according to $\tilde{S}_{i,t} = \tilde{S}_{i,t-1} - \tilde{I}_{i,t} /\rho$, where *ρ* is the probability that a person infected with DENV reported to the Health Department. Although there is a great deal of variability in *ρ* due to variation in rates of symptomatic disease and health-seeking behavior in different populations, we adopted a value of $\rho= 0.18$ based on a recent meta-analysis [10]. This parameter accounts for the fact that many DENV infections are mild or asymptomatic, which is important when tracking the susceptible population due to the fact that individuals exposed to DENV become immune thereafter regardless of the extent to which they experience symptoms. One complication that we did not account for due to a lack of data is that there are four distinct DENV serotypes, with long-lasting immunity being specific only to the serotype(s) to which one has been exposed. There is, however, a short-term period of cross-immunity that is protective against all serotypes following exposure to only a single serotype, with the duration of this period (maximum-likelihood estimate: 1.88 y, 95% confidence interval: 0.88–4.31 y [43]) being similar to the timescale of our data set as a whole.

Following the standard form of TSIR models, we assumed that new cases among people spending time in town *i* were acquired on week *t* according to

1 $$ \tilde{I}_{i,t} = \beta_{i} ( t ) \frac{\tilde{I} '_{i,t}}{ \tilde{N}_{i}} \tilde{S} '_{i,t}, $$

where $\beta_{i} ( t )$ is the transmission coefficient in town *i* at time *t*. The prime notation for $\tilde{I} '_{i,t}$ and $\tilde{S} '_{i,t}$ denotes the numbers of infected and susceptible people in the “generation” prior to *t*. The obligatory role of a mosquito in the transmission of DENV from one person to another is associated with a relatively long generation interval compared to directly transmitted pathogens. Whereas most TSIR models treat consecutive time steps as distinct generations, we obviated the need to temporally aggregate the data to such a large extent by calculating

2 $$ \tilde{I} '_{i,t} = \sum_{n=1}^{5} \omega_{n} \tilde{I}_{i,t-n}, $$

where

3 $$ \omega_{n} = \frac{1}{F ( 35 )} \biggl( \frac{1}{7} \int_{7(n-1)}^{7n} F ( \tau+7 ) -F ( \tau)\, d\tau\biggr) $$

is the probability that a case in week *t* is attributable to a case that occurred in week $t-n$ as defined by a generation interval with distribution function *F* [38]. We adopted a distribution function estimated by Siraj et al. [52] at a temperature of 30°C (the average daily temperature in Lahore during 2011–2014), which resulted in values of $\omega_{n}$ of $4.8 \times10^{-4}, 0.168, 0.440, 0.267$, and 0.125 for $n = 1, \dots, 5$, respectively. $\tilde{S} '_{i,t}$ was calculated similarly.

*Model fitting*: A primary advantage of the TSIR framework is that it allows for a model to be fitted to incidence data using regression techniques, which are easier to implement than alternative approaches to fitting dynamic models to time series data. To do so, we took the natural log of Eq. (1) and rearranged to obtain the regression equation

4 $$ \ln( \tilde{I}_{i,t} ) - \ln\bigl( \tilde{S} '_{i,t} \bigr) + \ln( \tilde{N}_{i} ) = \ln\bigl( \beta_{i} ( t ) \bigr) + \ln\bigl( \tilde{I} '_{i,t} \bigr) + \epsilon_{t}, $$

where

5 $$ \ln\bigl( \beta_{i} ( t ) \bigr) = s_{\mathrm{secular},i} ( t ) + s_{\mathrm{seasonal},i} \bigl( t-52 \lfloor t/52 \rfloor\bigr) $$

and each $\epsilon_{t}$ is an independent and identically distributed normal random variable. We posed $\ln ( \beta_{i} ( t ) )$ as a shape-constrained additive model (SCAM) and estimated parameters describing its two components using the scam function in the scam package [41] in R [42]. To prevent data points near the beginning and end of the time series from leading to unreasonably large values of $\beta_{i} ( t )$ when extrapolating beyond those data points, we constrained $s_{\mathrm{secular},i}$ to be a concave function. We modeled $s_{\mathrm{seasonal},i}$ as a cyclic cubic spline to ensure that its values at the beginning and end of the year were equal up to their second derivative. Under all mobility assumptions other than ideal free, we estimated separate town-specific functions for each of the two components of $\ln ( \beta_{i} ( t ) )$. Under the ideal free mobility assumption, we estimated only a single $\ln ( \beta ( t ) )$ that applied to all towns due to the fact that the mobility-transformed data were strictly proportional to each other under this assumption.

## Results

**Figure 1:**
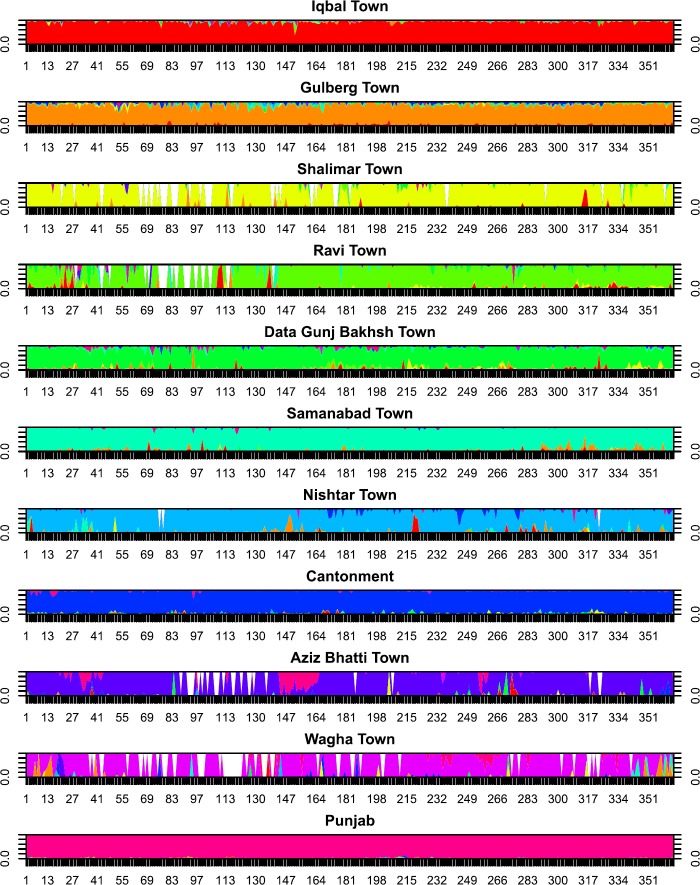
Relative proportion of tweets made in the town indicated in the panel label by residents of every other town. Different colors represent different home location of residents

*Human mobility data*: Tweet-derived movement estimates showed relatively low movement outside the town of residence, with the mean proportion of time spent within one’s town of residence being 91.2% (range: 84.0–96.8%). The town from which the largest proportion of non-resident tweets was made was Gulberg Town (1.7%), and the fewest were made in Wagha Town (0.16%). Although there was substantial day-to-day variation in Twitter activity across towns (Fig. 1), the extent to which that variation was driven by a set of deterministic factors or sampling noise was not apparent. Based on the limited sample of tweets available and their incomplete coverage over the study period, we used the time-averaged proportion of tweets by residents of each town made in every other town in the epidemiological analysis.

**Figure 2:**
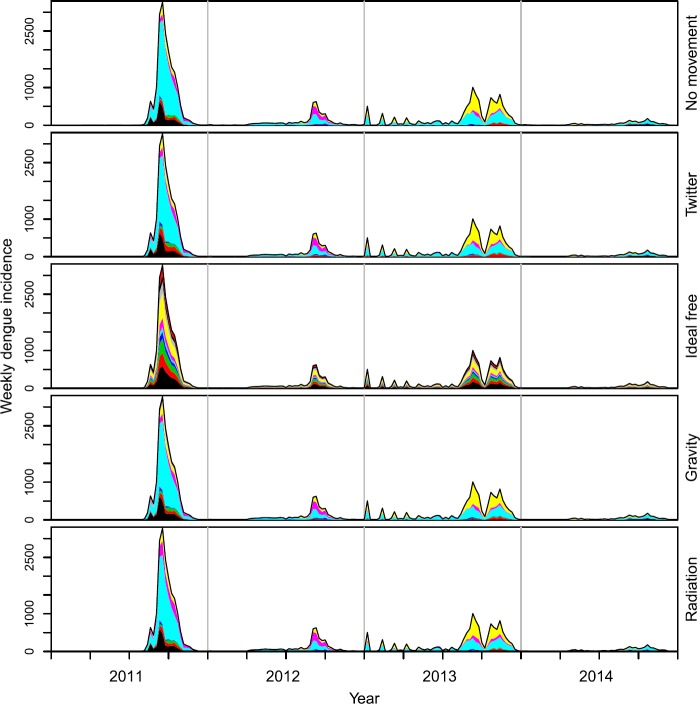
Time series stratified by towns in which cases were assumed to be acquired (colors) under five different assumptions about mobility among towns (rows)

**Figure 3:**
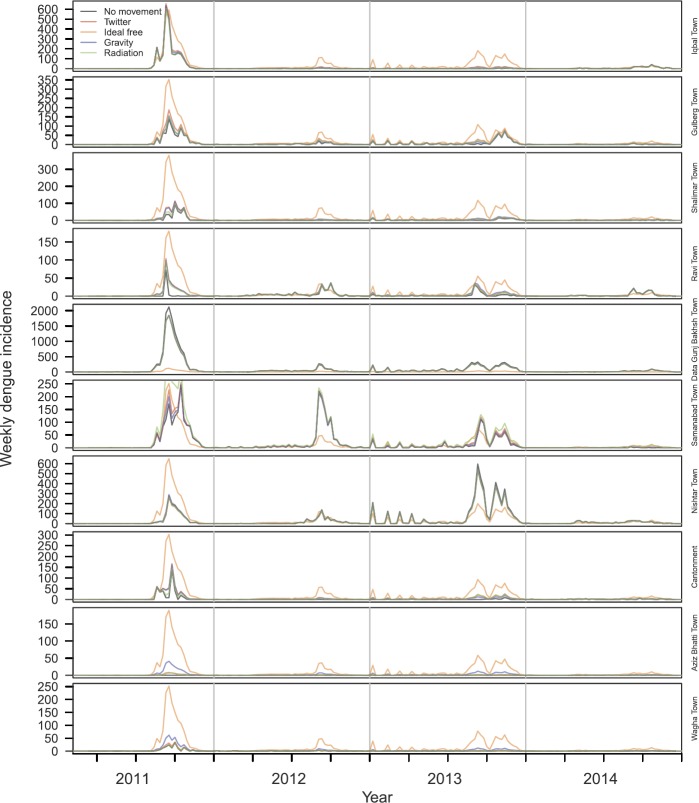
Time series stratified by towns in which cases were assumed to be acquired (rows) under five different assumptions about mobility among towns (line type)

*Mobility-based transformation of incidence data*: Applying the five mobility matrices to dengue incidence time series stratified by town of residence, we obtained notably different time series of the towns in which the cases were acquired. Under the assumption of no movement outside one’s town of residence, the residence-based and mobility-based time series were identical. Under the assumption that mobility follows Twitter, gravity, or radiation movement patterns, the mobility-based time series was mostly similar to the residence-based time series (Fig. 2), although redistribution from high-incidence towns to low-incidence towns was visually apparent (Fig. 3). This redistribution was attributable to the partially homogenizing effect of inter-town mobility. Under the assumption that mobility follows an ideal-free distribution, the mobility-based time series was stratified proportional to town population size (Fig. 2), resulting in time series that followed identical dynamics (Fig. 3). Whereas the distribution of incidence across towns was temporally constant under the ideal-free assumption, there was substantially more temporal variation in the distribution of incidence across towns under the Twitter, gravity, radiation, and no-movement assumptions (Fig. 2).

**Figure 4:**
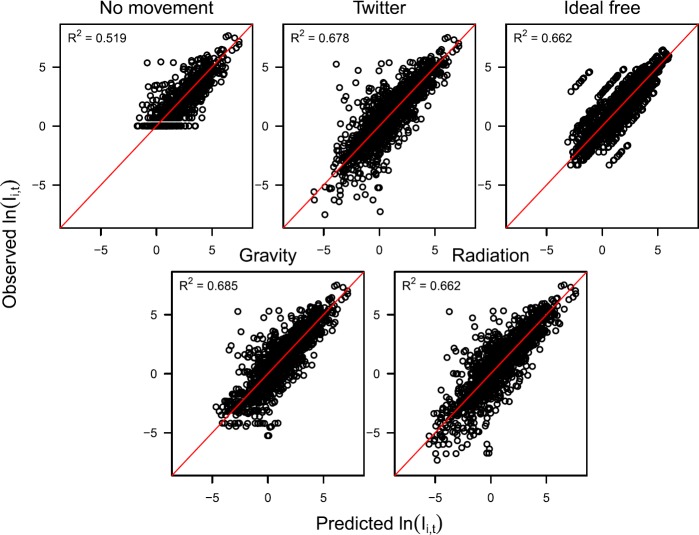
Relationships between predicted (*x*-axis) and observed (*y*-axis) log incidence based on models fitted to five different mobility-based incidence time series (panels). The coefficient of determination, $R^{2}$, associated with each best-fit model is indicated in each panel. Values of observed incidence vary across panels due to the effect of different assumptions about mobility used to transform the residence-based time series to mobility-based time series. For example, log incidence under the assumption of no movement never falls below 0, because there were no fractional cases observed in the raw data. Fractional incidence did occur in the other two time series due to each person’s incidence of disease being partitioned across the towns proportional to assumed mobility patterns. Under the ideal free assumption, the diagonal sets of points are a result of incidence on a given day varying across towns only in proportion to their different population sizes

*Model fitting*: Best-fit models to all five mobility-based time series explained a relatively high proportion of variation in incidence, with the coefficient of determination, $R^{2}$, ranging 0.519–0.685 (Fig. 4). In general, the time series data was explained similarly well by each of the models that performed a mobility transformation; i.e., Twitter ($R^{2}=0.678$), ideal free ($R^{2}=0.662$), gravity ($R^{2}=0.685$), and radiation ($R^{2}=0.662$). The data was explained less well by the model that assumed no movement ($R^{2}=0.519$). Rather than an indication of the inadequacy of the no movement assumption, we interpreted this lower $R^{2}$ value as a consequence of the fact that $\tilde{I}_{i,t}$ in Eq. (1) is integer-valued under the no movement assumption and continuous under the other mobility assumptions. Because our model’s generation-interval adjustment in Eq. (2) results in $\tilde{I} '_{i,t}$ being continuous, the mobility assumptions associated with continuous values of $\tilde{I}_{i,t}$ have an inherent advantage in fitting the data, especially for $\tilde{I} '_{i,t} <1$ (Fig. 4). As a result, comparison of $R^{2}$ values calculated in reference to the mobility-based time series to which the models were fitted indicates no clear distinction among the mobility assumptions and their appropriateness for modeling the mobility-based time series.

**Figure 5:**
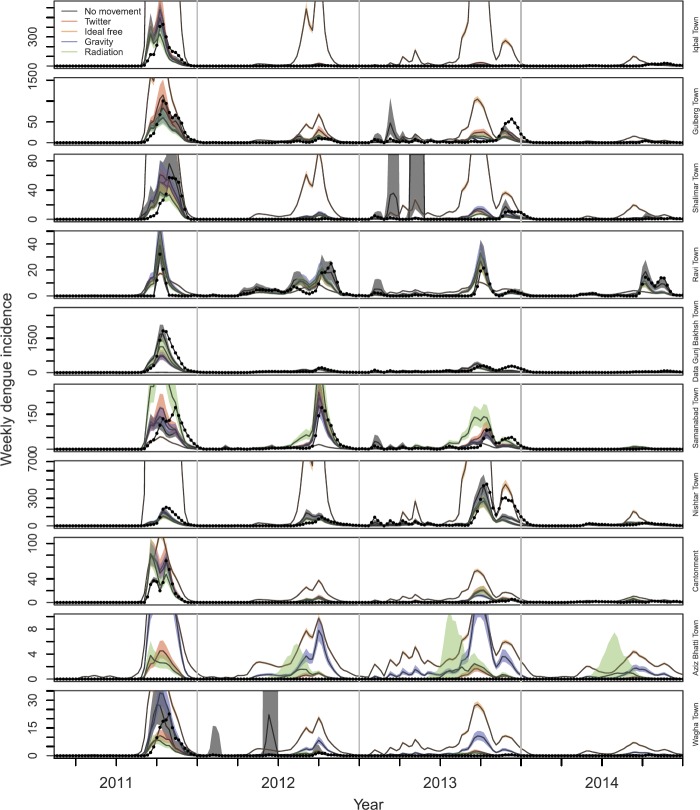
Predicted values of residence-based incidence in each town (rows) using the best-fit model under each of five different assumptions about mobility (colors). Observed values of residence-based incidence in each town are shown with black dots, and bands show 95% confidence intervals on model predictions

Another way that we examined model fit was based on how well each best-fit model matched the original time series once a model’s one-step ahead predictions of $\tilde{I}_{i,t}$ were transformed back to predictions of $I_{j,t}$ using the *H* matrix under which a given model was fitted. Under the no movement assumption, $\tilde{I}_{i,t}$ and $I_{j,t}$ were, by definition, the same. These predictions were generally consistent with the data, although there were instances in Gulberg Town, Shalimar Town, and Wagha Town in which model predictions far exceeded the data during certain time periods (Fig. 5). This was likely due to the seasonal component of $\beta_{i} ( t )$ being influenced too heavily by years with larger outbreaks. Under the Twitter, gravity, and radiation assumptions, predictions of $I_{j,t}$ were often similar to or nearly as good as predictions based on the model fitted under the no movement assumption (Fig. 5). These models performed less well in the towns with the lowest incidence—i.e., Aziz Bhatti Town and Wagha Town—due to those models’ predictions of a greater degree of imported incidence from towns with high transmission than actually occurred (Fig. 5). In terms of ability to predict $I_{j,t}$, the model fitted under the ideal free assumption performed the worst by far. In the towns with the greatest incidence per capita, this model predicted either too few cases overall (Samanabad Town) or incidence patterns that were not as peaked as was observed locally (Ravi Town) (Fig. 5). In all other towns, the model fitted under the ideal free assumption overpredicted incidence, sometimes by several hundred cases in a single week (e.g., Iqbal Town, Nishtar Town) (Fig. 5).

**Figure 6:**
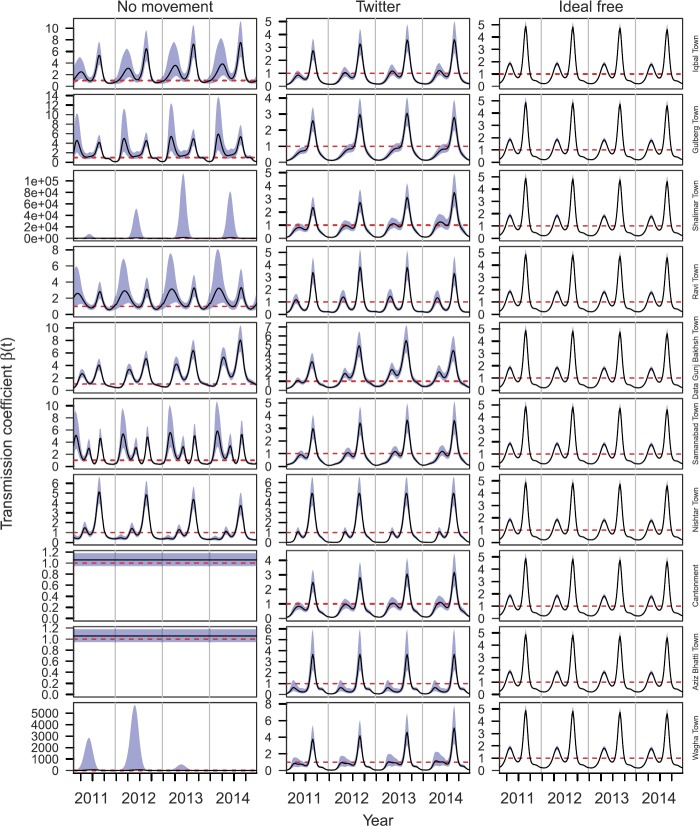
Temporal variation in $\beta_{i} (t)$ for different towns (rows) under three different mobility assumptions (columns): no movement (left), Twitter (center), and ideal free (right). The black line shows the mean of the best-fit model and blue bands show standard error around the mean. The dashed red line indicates where $\beta_{i} ( t ) =1$

**Figure 7:**
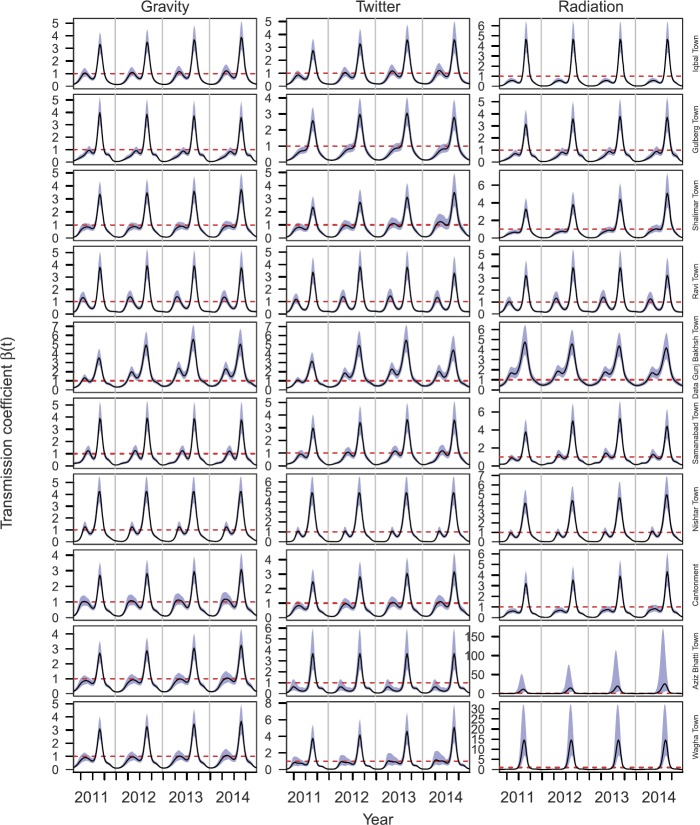
Temporal variation in $\beta_{i} (t)$ for different towns (rows) under three different mobility assumptions (columns): gravity model (left), Twitter (center), and radiation model (right). The black line shows the mean of the best-fit model and blue bands show standard error around the mean. The dashed red line indicates where $\beta_{i} ( t ) =1$

*Transmission model inferences*: Inferences of $\beta_{i} (t)$ under different mobility assumptions varied widely. Variation in $\beta_{i} (t)$ across towns was maximized under the assumption of no movement, with patterns ranging from nearly flat in Cantonment and Aziz Bhatti Town to a single seasonal peak in Shalimar Town and Wagha Town to multiple annual peaks of different heights across years in the other towns (Fig. 6, left). Under the no movement assumption, confidence intervals for $\beta_{i} (t)$ were unreasonably high in Shalimar Town and Wagha Town (Fig. 6, left). By design, inferences of $\beta_{i} (t)$ under the ideal free assumption were identical across towns and displayed a pattern of two seasonal peaks, with the one in the third quarter being larger (Fig. 6, right). This same general pattern was apparent in the inferences of $\beta_{i} (t)$ under the Twitter mobility assumption, but there was clear variability across towns, with differences in the heights of the peaks, their timing, and other aspects of their shape (Fig. 6, center). Inferences of $\beta _{i} (t)$ under the gravity and radiation mobility assumptions were similar to those under the Twitter assumption, with gravity being extremely similar (Fig. 7, left) and radiation being similar in most towns but having considerably larger peaks in Aziz Bhatti Town and Wagha Town (Fig. 7, right).

**Figure 8:**
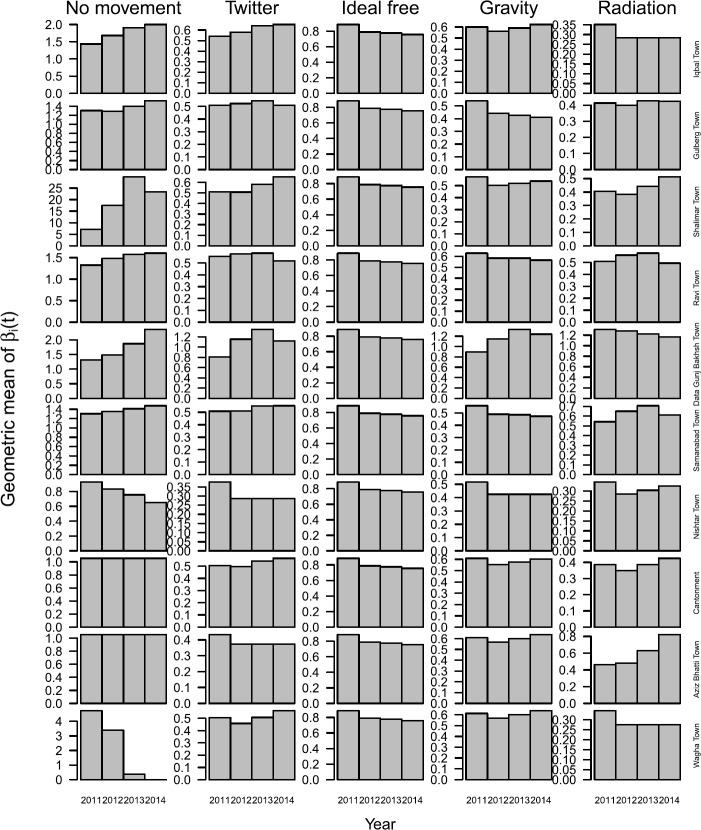
Geometric means of $\beta_{i} (t)$ stratified by year (x-axis), town (rows), and mobility assumption (columns)

**Figure 9:**
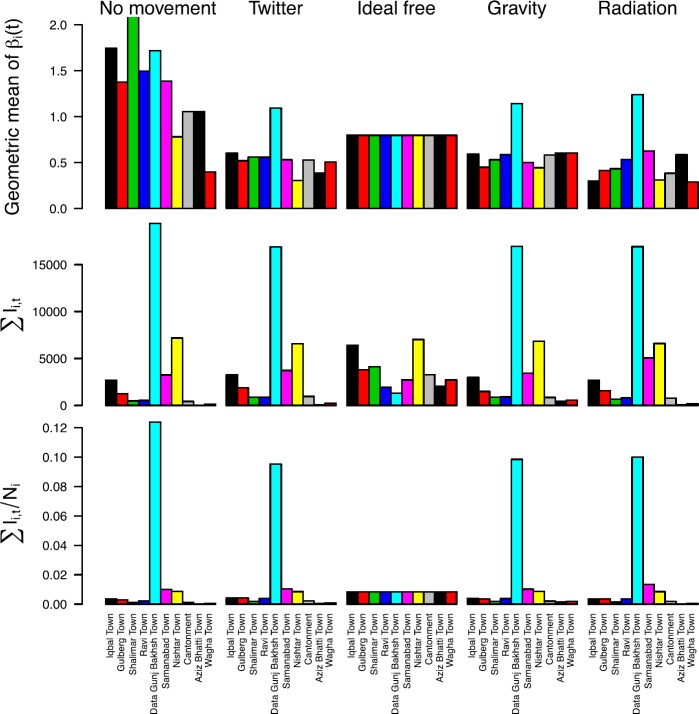
Measures of transmission and incidence across towns (colors) under different mobility assumptions (columns) aggregated over the entire 2011–2014 time period. The upper left y-axis was cut off to permit viewing of the majority of values; the geometric mean of $\beta_{i} (t)$ for Shalimar town is 17.49

A general tendency for variation in $\beta_{i} (t)$ across towns under different assumptions about mobility was reinforced by examining geometric means of $\beta_{i} (t)$ over time. Under the ideal free mobility assumption, the geometric mean of $\beta_{i} (t)$ decreased every year (Fig. 8). In contrast, the geometric mean of $\beta_{i} (t)$ was greater in 2014 than in 2011 in approximately half the towns under the Twitter, gravity, and radiation mobility assumption, with differences across models in terms of which towns experienced those increases (Fig. 8). The degree of inter-annual variation in the geometric mean of $\beta_{i} (t)$ was greatest under the no movement assumption, moderate under the Twitter, gravity, and radiation assumptions, and least under the ideal free assumption (Fig. 8). Under the Twitter, gravity, and radiation assumptions, the geometric mean of $\beta_{i} (t)$ across all years was highest in Data Gunj Bakhsh Town, which was also highest in both absolute and per capita terms for both residence-based and mobility-based incidence (Fig. 9). Otherwise, there was little correspondence between the geometric mean of $\beta_{i} (t)$ and either absolute or per capita incidence of the other nine towns under the Twitter, gravity, and radiation mobility assumptions. There was also no clear correspondence between $\beta_{i} (t)$ and incidence under the no movement assumption, and both the geometric mean of $\beta_{i} (t)$ and per capita incidence were equal across towns under the ideal free assumption, as expected (Fig. 9).

## Discussion

Urban areas exhibit spatial heterogeneity in numerous factors that are relevant to infectious disease transmission, which can contribute to spatial variation in transmission [2] and interact with temporal drivers of transmission [44]. Our work contributes to understanding of infectious disease dynamics in urban settings by highlighting the important role that human mobility plays in relating observed patterns of disease incidence to inferred patterns of disease transmission. On the one hand, our results show that assuming that human mobility is well mixed at the scale of a city (ideal free assumption) fails to capture underlying spatial heterogeneity in transmission and can lead to incorrect conclusions about secular trends in transmission across years. In some ways, this behavior is not surprising, but a systematic review of the literature on mathematical modeling of mosquito-borne disease transmission showed that this assumption is extremely prevalent across this field [45]. On the other hand, assuming that different districts of a city are isolated (no movement assumption) may lead to exaggerated and biologically unrealistic inferences about transmission patterns. Whether it be Twitter or other data streams [3], incorporating realistic patterns of human mobility among districts of a city may help strike an appropriate balance between the tendencies of these two extreme assumptions. Regardless of whether such data streams provide a “correct” picture of mobility, it is encouraging that our results showed that Twitter, gravity, and radiation assumptions all resulted in similar epidemiological inferences.

The results of our analysis are a useful case study for any infectious disease, but they have particular importance for dengue. First, dengue mitigation strategies tend to be spatially reactive to reported incidence [59]. Other than the town with the highest per capita incidence, we did not identify a strong correspondence between towns with the highest incidence and those with the highest inferred transmission coefficients (similar to recent findings for malaria [11]), which suggests that reactive control deployed to areas with the highest incidence may not necessarily have as much impact on reducing transmission as more optimized strategies might. Second, due to adverse outcomes associated with vaccinating individuals with no prior DENV exposure with the only currently licensed dengue vaccine [20], it is recommended that this vaccine only be used in areas with high transmission intensity [17]. Although consideration is given to subnational variation in transmission intensity (see https://mrcdata.dide.ic.ac.uk/_dengue/dengue.php), our results indicate that this issue may also warrant attention at intra-urban scales. Third, the increasing trend in the transmission coefficient that we observed under the Twitter mobility assumption serves as a reminder that a decreasing trend in incidence may not be indicative of a decreasing trend in factors underlying transmission. Instead, it appears that incidence has probably decreased due to an increase in herd immunity and that conditions remain ripe for transmission, which could result in a large epidemic once a sufficient number of susceptibles build up from births and waning heterotypic immunity.

Although our Twitter mobility assumption may be an improvement over some of the other assumptions that we examined, there are a number of other considerations about intra-urban human mobility that are likely to affect DENV transmission. We have limited understanding of how representative Twitter patterns are compared to actual movements of people and see them more as an approximation to relative movements between towns. At the same time, Twitter data have the advantage of being widely accessible for many urban areas worldwide, whereas alternative models for intra-urban human mobility tend to have been developed around specific settings (e.g., [37]). In addition, there may be interactions between disease symptoms, infectiousness, and mobility in DENV-infected people [13, 39] that complicate the assumption that tweets by presumably healthy people are a suitable approximation of mobility patterns of people involved in transmission [58]. Higher order descriptions of movement may also be necessary to accurately capture transmission dynamics, as social network structure has been shown to affect transmission dynamics in urban environments [46, 54, 57]. There is also the perennial question of what spatial scale is satisfactory for modeling infectious disease dynamics [36, 40]. Examinations of intra-urban DENV transmission patterns in Bangkok, Thailand suggest that there can be strong spatial heterogeneities of relevance to transmission dynamics at scales as small as hundreds of meters [47, 49].

Overall, our results showed that the inferred degree of variation in a spatially and temporally variable transmission coefficient was sensitive to five different assumptions about intra-urban mobility that we considered. This approach extends previous applications of the TSIR model that estimated either seasonally varying transmission coefficients according to pre-defined functions (e.g., [16]) or completely independent values of the transmission coefficient at each time step (e.g., [35]) by blending the same underlying conceptual approach with a powerful new regression technique [41] and applying it in a spatial context. Although our analysis does not account for specifically which factors underlie variation in the transmission coefficient that we uncovered, there are many well-known candidates that could be incorporated into future analyses [7, 8, 25, 52]. Either way, we expect that our results about the sensitivity of transmission inferences to assumptions about intra-urban mobility would still apply. More generally, we hope that this case study will serve as a guiding example to the growing number of data scientists engaging in analyses of infectious disease dynamics. The increasing availability of data from Twitter and other Internet-based streams provide an exciting opportunity for extracting new understanding from time series of infectious disease incidence, if used within an appropriate conceptual framework.

## Electronic Supplementary Material

Below is the link to the electronic supplementary material.

Supplementary information (PDF 109 kB)
