# Supplementary material for: Inferences about spatiotemporal variation in dengue virus transmission are sensitive to assumptions about human mobility: a case study using geolocated tweets from Lahore, Pakistan
Source: EPJ Data Sci. 2018 Jun 11;7(1):16. doi: 10.1140/epjds/s13688-018-0144-x (PMC6404370; doi:10.1140/epjds/s13688-018-0144-x)
Supplement: Supplementary file 1 — Supplementary information (PDF 109 kB) [file 13688_2018_144_MOESM1_ESM.pdf]

Supplementary information

Inferences about spatiotemporal variation in dengue virus transmission are sensitive to assumptions about human mobility: a case study using geolocated tweets from Lahore, Pakistan

Kraemer, M.U.G.[1,2,3,§], Bisanzio, D.[4,5], Reiner, R.C.[6], Zakar, R.[7], Hawkins, J.B.[1,2], Freifeld, C.C.[2,8], Smith, D.L.[5,9], Hay, S.I.[6], Brownstein, J.S.[1,2], Perkins, T.A.[10,§]

1. Department of Pediatrics, Harvard Medical School, Boston, MA, USA
2. Computational Epidemiology Lab, Boston Children's Hospital, Boston, MA, USA
3. Department of Zoology, University of Oxford, Oxford, UK
4. RTI International, Washington D.C., USA
5. Center for Tropical Diseases, Sacro Cuore-Don Calabria Hospital, Negrar, Verona, Italy
6. Institute for Health Metrics and Evaluation, University of Washington, Seattle, WA, USA
7. Department of Public Health, University of Punjab, Lahore, Pakistan
8. College of Computer and Information Science, Northeastern University, Boston, MA, USA
9. Sanaria Institute for Global Health and Tropical Medicine, Rockville, MD, USA
10. Department of Biological Sciences and Eck Institute for Global Health, University of Notre Dame, Notre Dame, IN, USA

[§]**Authors of correspondence**:

Moritz UG Kraemer, DPhil
Computational Epidemiology Lab, Harvard University
300 Longwood Ave
MA, 02115 Boston
kramer.moritz@gmail.com

T. Alex Perkins, PhD
347 Galvin Life Science Center
University of Notre Dame
Notre Dame, IN 46556
taperkins@nd.edu

Table S1: Human mobility metric as per gravity model.

	Iqbal.Town	Gulberg.Tow	Shalimar.To	Ravi.Town	Data.Gunj.Ba	Samanabad.	Nishtar.Tow	Cantonment	Aziz.Bhatti.T	Wagha.Tow
Iqbal Town	0.95071151	0.00553099	0.00552735	0.00540811	0.00535359	0.00548256	0.00563958	0.00549486	0.00540688	0.00544458
Gulberg Tow	0.01798011	0.84055491	0.01789719	0.01746902	0.01736762	0.01777666	0.01808941	0.01782643	0.01748305	0.01755561
Shalimar To	0.00698087	0.00695325	0.93801586	0.00684688	0.00675732	0.00687344	0.00701365	0.00690607	0.00681309	0.00683956
Ravi Town	0.01169284	0.01161859	0.01172127	0.89608667	0.01133526	0.01151933	0.01172971	0.01152367	0.0113571	0.01141554
Data Gunj Ba	0.01503785	0.01500693	0.01502876	0.01472645	0.86610123	0.01490343	0.01509262	0.01483634	0.0145989	0.0146675
Samanabad	0.00771717	0.00769725	0.00766048	0.00749942	0.00746827	0.9316431	0.0077417	0.00759779	0.00746824	0.00750658
Nishtar Tow	0.01117074	0.01102224	0.01099984	0.01074603	0.01064287	0.01089423	0.90188411	0.01098066	0.0107906	0.01086868
Cantonment	0.0054531	0.00544205	0.00542657	0.00528937	0.00524171	0.00535673	0.0055015	0.9515885	0.00534334	0.00535714
Aziz Bhatti T	0.00364477	0.00362536	0.00363642	0.00354092	0.0035035	0.00357657	0.00367226	0.00362951	0.96756191	0.00360877
Wagha Tow	0.01343995	0.01333092	0.01336808	0.01303334	0.01288987	0.01316439	0.01354489	0.01332537	0.01321507	0.88068812

Table S2: Human mobility metric as per radiation model.

	Iqbal.Town	Gulberg.Tow	Shalimar.To	Ravi.Town	Data.Gunj.Ba	Samanabad.	Nishtar.Tow	Cantonment	Aziz.Bhatti.T	Wagha.Tow
Iqbal Town	0.95071151	0.00464972	0.00200325	0.00117877	0.00121689	0.01829407	0.01878077	0.00246325	0.00042652	0.00027527
Gulberg Tow	0.00424727	0.84055491	0.01661344	0.00506369	0.01748131	0.07435086	0.00946928	0.02556011	0.00422534	0.00243381
Shalimar To	0.00180863	0.01333619	0.93801586	0.02248866	0.00847237	0.00504835	0.00320064	0.0030844	0.00267679	0.00186811
Ravi Town	0.00133662	0.00580199	0.03670333	0.89608667	0.04428317	0.00778463	0.00236535	0.00292794	0.00132972	0.00138058
Data Gunj Ba	0.00113956	0.01189648	0.00553933	0.01477773	0.86610123	0.09372208	0.00254065	0.00249626	0.00113368	0.000653
Samanabad	0.00149959	0.02425299	0.00609667	0.00510505	0.02413782	0.9316431	0.00279628	0.00274742	0.00124774	0.00047334
Nishtar Tow	0.01410225	0.02256662	0.00362631	0.00141646	0.00168172	0.00984524	0.90188411	0.03886225	0.00300866	0.00300639
Cantonment	0.00108793	0.01367122	0.00594785	0.00043325	0.00118476	0.00187855	0.00226485	0.9515885	0.02057106	0.00137202
Aziz Bhatti T	0.00044094	0.00138345	0.00298071	0.00031324	0.00033569	0.00055426	0.00078032	0.0211542	0.96756191	0.00449528
Wagha Tow	0.00219591	0.00688961	0.01484398	0.00155993	0.00167172	0.0027602	0.00388598	0.0299518	0.05555274	0.88068812

Table S3: Human mobility metric as per Twitter.

	Iqbal Town	Gulberg Tow	Shalimar To	Ravi Town	Data Gunj Ba	Samanabad	Nishtar Tow	Cantonment	Aziz Bhatti T	Wagha Tow
Iqbal Town	0.95071151	0.00923713	0.00160491	0.005849	0.00415493	0.01242912	0.00896965	0.00608082	0.00037448	0.00058847
Gulberg Tow	0.02893194	0.84055491	0.010473	0.0045634	0.02464234	0.02411755	0.01473977	0.04581651	0.00419833	0.00196226
Shalimar To	0.00712089	0.00954847	0.93801586	0.01650753	0.02168636	0.00161838	0.00064735	0.00113287	0.00226574	0.00145655
Ravi Town	0.02785762	0.00630113	0.01746628	0.89608667	0.03526421	0.01249171	0.00055273	0.00243201	0.00121601	0.00033164
Data Gunj Ba	0.0237955	0.0249735	0.02041858	0.01821966	0.86610123	0.02352063	0.00192406	0.01390034	0.00290572	0.00424078
Samanabad	0.01270553	0.04004254	0.00054617	0.0016385	0.00787628	0.9316431	0.00077613	0.00419685	0.00051742	5.75E-05
Nishtar Tow	0.02230715	0.02186278	0.00053324	0.00017775	0.00071098	0.02292926	0.90188411	0.02701742	0.00106648	0.00151084
Cantonment	0.00987561	0.01019079	0.00552614	0.00027316	0.00542108	0.00552614	0.0042234	0.9515885	0.00487477	0.00250042
Aziz Bhatti T	0.00435996	0.00331357	0.00261597	0.0001744	0.00697593	0.00069759	0.0001744	0.0104639	0.96756191	0.00366236
Wagha Tow	0.00055494	0.04883463	0.00527192	0	0.00388457	0.02219756	0.00305216	0.0099889	0.02552719	0.88068812

Table S4: Human mobility metric assuming ideal free movement.

	Iqbal Town	Gulberg Tow	Shalimar To\	Ravi Town	Data Gunj Ba	Samanabad	Nishtar Towı	Cantonment	Aziz Bhatti T	Wagha Towr
Iqbal Town	0.18088826	0.10763951	0.11670947	0.05504058	0.03710825	0.07671147	0.19862344	0.09236305	0.05798555	0.07693042
Gulberg Tow	0.18088826	0.10763951	0.11670947	0.05504058	0.03710825	0.07671147	0.19862344	0.09236305	0.05798555	0.07693042
Shalimar To\	0.18088826	0.10763951	0.11670947	0.05504058	0.03710825	0.07671147	0.19862344	0.09236305	0.05798555	0.07693042
Ravi Town	0.18088826	0.10763951	0.11670947	0.05504058	0.03710825	0.07671147	0.19862344	0.09236305	0.05798555	0.07693042
Data Gunj Ba	0.18088826	0.10763951	0.11670947	0.05504058	0.03710825	0.07671147	0.19862344	0.09236305	0.05798555	0.07693042
Samanabad	0.18088826	0.10763951	0.11670947	0.05504058	0.03710825	0.07671147	0.19862344	0.09236305	0.05798555	0.07693042
Nishtar Towı	0.18088826	0.10763951	0.11670947	0.05504058	0.03710825	0.07671147	0.19862344	0.09236305	0.05798555	0.07693042
Cantonment	0.18088826	0.10763951	0.11670947	0.05504058	0.03710825	0.07671147	0.19862344	0.09236305	0.05798555	0.07693042
Aziz Bhatti T	0.18088826	0.10763951	0.11670947	0.05504058	0.03710825	0.07671147	0.19862344	0.09236305	0.05798555	0.07693042
Wagha Towr	0.18088826	0.10763951	0.11670947	0.05504058	0.03710825	0.07671147	0.19862344	0.09236305	0.05798555	0.07693042

Table S5: Human mobility metric as per no movement assumption.

	Iqbal Town	Gulberg Tow	Shalimar To\	Ravi Town	Data Gunj Ba	Samanabad	Nishtar Towı	Cantonment	Aziz Bhatti T	Wagha Towr
Iqbal Town	1	0	0	0	0	0	0	0	0	0
Gulberg Tow	0	1	0	0	0	0	0	0	0	0
Shalimar To\	0	0	1	0	0	0	0	0	0	0
Ravi Town	0	0	0	1	0	0	0	0	0	0
Data Gunj Ba	0	0	0	0	1	0	0	0	0	0
Samanabad	0	0	0	0	0	1	0	0	0	0
Nishtar Towı	0	0	0	0	0	0	1	0	0	0
Cantonment	0	0	0	0	0	0	0	1	0	0
Aziz Bhatti T	0	0	0	0	0	0	0	0	1	0
Wagha Towr	0	0	0	0	0	0	0	0	0	1

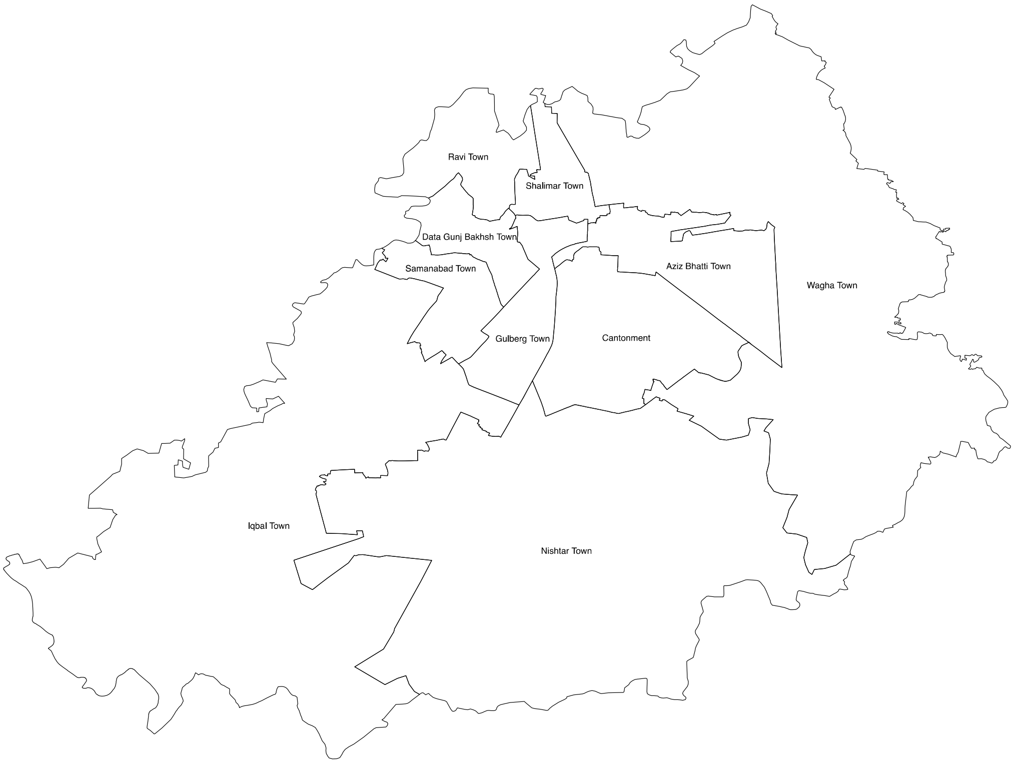

Supplementary Figure 1. Map of towns in Lahore.
